# Mercury accumulation and biomarkers of exposure in two popular recreational fishes in Hawaiian waters

**DOI:** 10.1007/s10646-023-02684-1

**Published:** 2023-07-25

**Authors:** Stephanie Shaw Holbert, Colleen E. Bryan, Keith E. Korsmeyer, Brenda A. Jensen

**Affiliations:** 1 College of Natural and Computational Sciences, Hawaii Pacific University, Kaneohe, HI USA; 2 Chemical Sciences Division, National Institute of Standards and Technology, Charleston, SC USA

**Keywords:** Mercury, Metallothionein, Thioredoxin reductase, Bonefish, Trevally, mRNA expression

## Abstract

Mercury (Hg) exposure has not been examined in many recreational nearshore fish species that are commonly consumed around the Hawaiian Islands. Specific gene transcripts, such as metallothionein (MET) and thioredoxin reductase (TrxR), can be used to examine Hg exposure responses in aquatic organisms. This study measured total mercury (THg) in four species from two groups of Hawaiian nearshore fishes: giant trevally (*Caranx ignobilis*, *n* = 13), bluefin trevally (*C. melampygus*, *n* = 4), sharp jaw bonefish (*Albula virgata*, *n* = 2), and round jaw bonefish (*A. glossodonta*, *n* = 19). Total Hg accumulation and abundance profiles of MET and TrxR were evaluated for muscle, liver, and kidney tissues. Total Hg in round jaw bonefish and giant trevally tissues accumulated with length and calculated age. In round jaw bonefish tissues, mean THg was greater in kidney (1156 ng/g wet mass (wm)) than liver (339 ng/g wm) and muscle (330 ng/g wm). Giant trevally muscle (187 ng/g wm) and liver (277 ng/g wm) mean THg did not differ significantly. Fish species in this study were compared to commercial and local fish species with state and federal muscle tissue consumption advisories based on THg benchmarks developed by the U.S. Food and Drug Administration (FDA) and Environmental Protection Agency (EPA). Both bonefishes had mean muscle THg that exceeded benchmarks suggesting consumption advisories should be considered. MET transcript in round jaw bonefish kidney tissue and kidney THg exhibited a marginally significant positive correlation, while TrxR transcript in liver tissue negatively correlated with increasing liver THg. These results contribute to our understanding of Hg exposure associated health effects in fish.

## Introduction

Mercury (Hg) in fishes has been a concern for decades because of its capacity to accumulate in great, potentially toxic amounts, which may pose a health risk for higher trophic level fishes, humans, and other animals that consume fish (Alexander et al. 2004; Mergler et al. 2007; Scheuhammer et al. 2007). Mercury is known to bioaccumulate in organisms over time, and methylmercury (MeHg), the most toxic form of Hg, tends to biomagnify in mid to upper trophic level species (Mason et al. 1996; Fitzgerald and Clarkson 1991). Fishes take up both inorganic (Hg^2+^) and organic (MeHg) forms of Hg with MeHg usually making up more than 95% of the total mercury (THg) measured in fish muscle tissue (Bloom 1992; Bank et al. 2007; Sunderland 2007). In fish liver, MeHg can either be stored or demethylated into Hg^2+^ which is also stored in liver or transported to other tissues in the body (Wang et al. 2017; Pinzone et al. 2022). Kidney is the target organ for Hg^2+^ that has either been demethylated in other fish tissues or transported from the gills via water uptake (Baatrup et al. 1986). In Hawai‘i, fishing is important for sustenance, recreation, and livelihood. While several nearshore Hawaiian fish species are commonly caught for these purposes, few have been assessed for mercury.

Giant trevally (*Caranx ignobilis*), bluefin trevally (*Caranx melampygus*), sharp jaw bonefish (*Albula virgata*), and round jaw bonefish (*Albula glossodonta*) are popular tournament fish species found in the waters of Hawaiʻi around the Isle of O‘ahu. Trevallies and bonefishes play important but different roles in the trophic ecology of nearshore marine communities around Hawai‘i. Bonefishes have localized home ranges and diets that consists mainly of benthic invertebrates (Friedlander et al. 2008; Kamikawa et al. 2015). Adult sharp jaw bonefish have an average length of 45.2 centimeters (cm), while adult round jaw bonefish average 49.8 cm in length (Donovan et al. 2015). In contrast, trevally species are highly mobile predatory fishes that feed at higher trophic levels with diets of smaller fishes, crustaceans, and cephalopods (Meyer et al. 2001; Salini et al. 1994; Sudekum 1991; Major 1978). Bluefin trevally can reach sexual maturity at 35 cm and grow up to 80 cm, but giant trevally reach maturity around 60 cm and can grow to over 100 cm (Sudekum 1991; Pardee et al. 2021). These different ecological niches suggest that, compared with trevallies, bonefishes may be better indicator species because their localized home ranges would reflect local contamination levels in nearshore environments. Detectable Hg values and consumption advisories have been established for some popular, commercial pelagic fishes and some bottomfish species in Hawaiian waters, such as yellowfin tuna (*Thunnus albacares*) (Blum et al. 2013; Choy et al. 2009) and pink snapper (*Pristipomoides filamentosus*) (Sackett et al. 2015). However, Hg in giant trevally, bluefin trevally, sharp jaw bonefish, and round jaw bonefish are not well documented with no current consumption advisories in Hawai‘i.

Nearshore fish communities in Hawai‘i are exposed to local sources of Hg from storm-water runoff (Yamane and Lum 1985), groundwater discharge (Ganguli et al. 2014), and volcanic activity (Siegel and Siegel 1987). In addition, a steady increase in Asian anthropogenic Hg emissions over the past several decades has increased Hg deposition off the coast of Japan, thereby increasing concentrations in oceanic surface waters (Pacyna et al. 2006; Sunderland et al. 2009). These waters are laterally transported across the North Pacific in intermediate water masses to the eastern North Pacific (Sunderland et al. 2009). Drevnick and Brooks (2017) suggested the increase in Hg emissions from Asia is associated with a 5.5% per year increase of Hg in Pacific yellowfin tuna muscle from 1998 to 2008 and a 3.9% per year increase of Hg in Pacific bigeye tuna (*Thunnus obesus*) muscle from 2002 to 2008. If current deposition rates are maintained, Hg across the entire North Pacific Ocean basin may increase and subsequently affect contaminant burdens in pelagic and nearshore marine fishes around the Hawaiian Islands raising health concerns for people who depend on these fishes as food sources.

Elevated levels of Hg in fishes can cause negative effects on behavior (Vieira et al. 2009), reproduction (Drevnick and Sandheinrich 2003), feeding (Wiener and Spry 1996), and pathology (Branco et al. 2012; Hedayati et al. 2010). Metallothioneins (MET) are a protein family commonly recognized for their metal binding properties, regulating the homeostasis of essential metals, and protecting cells against non-essential metals, such as Hg (Hamer 1986; Kägi 1991; Piotrowski et al. 1974). Thioredoxin reductase (TrxR) is an important selenoenzyme in the thioredoxin system associated with many essential cellular functions including cellular stress responses and protection against oxidative damage (Schallreuter and Wood 1986; Schallreuter et al. 1986; Lillig and Holmgren 2007). Changes in liver and kidney MET mRNA expression and protein levels have been associated with waterborne and dietary Hg^2+^ and MeHg exposure in several fish species (Berntssen et al. 2004; Sinaie et al. 2010; Knapen et al. 2007). Inhibited TrxR activity has been the only associated change tested in fish species exposed to waterborne Hg^2+^ and MeHg with no known studies to date comparing TrxR mRNA expression to Hg exposure (Branco et al. 2011; 2012). These findings suggest the potential of MET and TrxR as biomarkers of Hg exposure in fish species.

The purpose of the present study was to 1) measure and compare THg in tissues (muscle, liver, and kidney) of four commonly consumed fish species of Hawai‘i, 2) evaluate species-specific relationships between fish length and THg, 3) compare muscle THg between study species and commercial pelagic fish species in order to provide relevant context for consumers, and 4) evaluate the relationship between fish THg and mRNA abundance profiles in liver and kidney of gene transcripts encoding proteins (TrxR and MET) that play an important role in cellular stress response and cellular metal homeostasis.

## Methods

### Sample collection

**Fig. 1:**
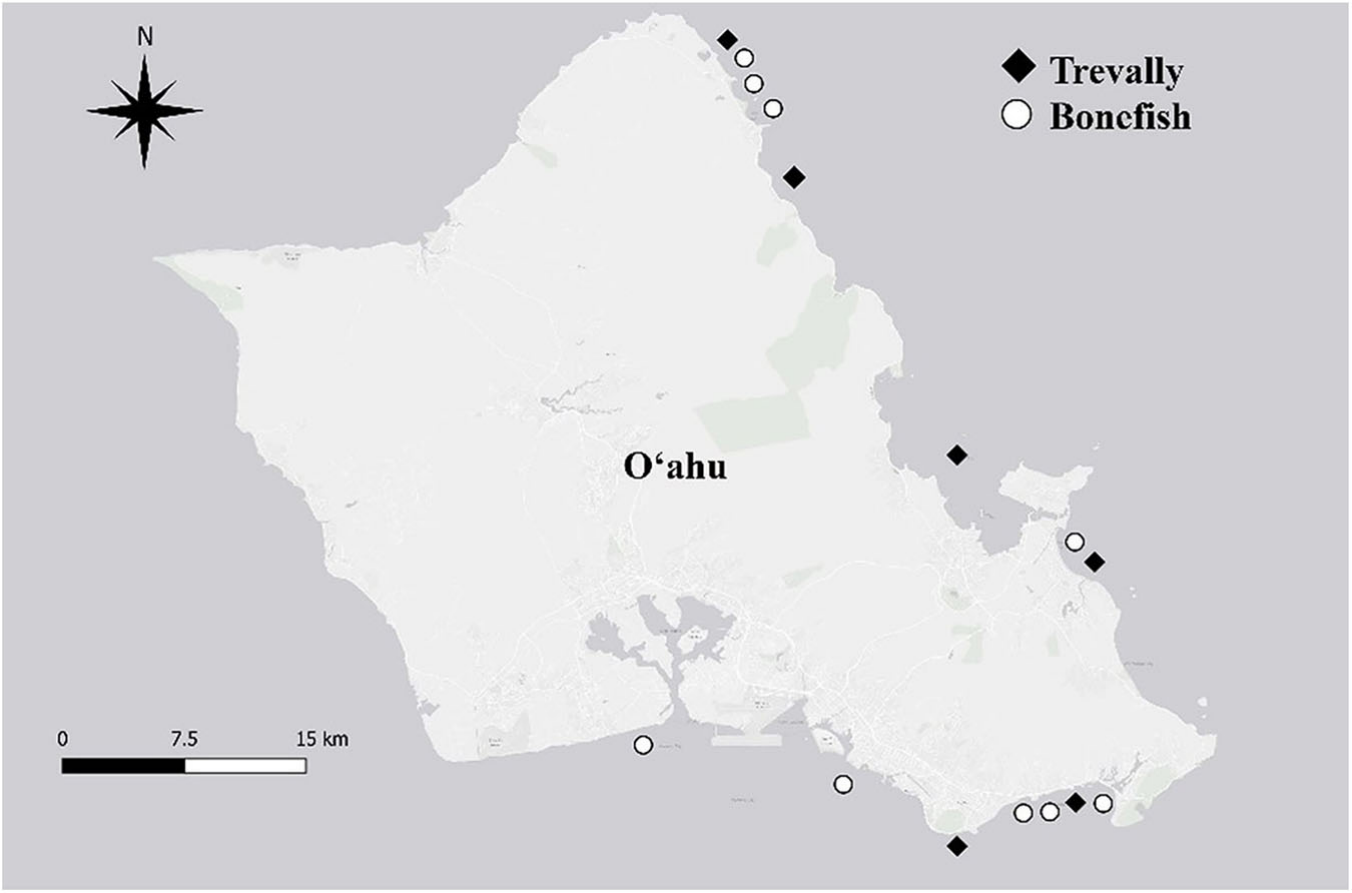
Isle of O‘ahu map showing catch locations for bonefish (white circles) and trevally (black diamonds) species samples during 2015 and 2016 fishing tournaments

Tissue samples were obtained from specimens of bluefin trevally (*n* = 4), giant trevally (*n* = 13), sharp jaw bonefish (*n* = 2), and round jaw bonefish (*n* = 19) collected opportunistically through donation by fishers at the Obake Shootout tournament November 8, 2015; the 11^th^ Annual Pat Hose Memorial ‘Ō‘io tournament November 15, 2015; and the KKC ‘Ō‘io Invitational tournament February 21, 2016. Length, sex, and approximate catch location (eastern and southern shores of O‘ahu) were recorded for each fish (Fig.1; Supplementary Information, SI, Tables S1 and S2). Sex was determined macroscopically before collection of other tissue. Sex was recorded as “unknown” if fish gonads appeared immature. Muscle tissue (sub-sample from below the left pectoral fin), whole liver, and whole kidney were collected from the two bonefish species, and liver and muscle tissue were collected from the two trevally species. Kidney tissue from the trevallies was too difficult to collect without damaging the fillets of the fish for the fishers contributing samples. Tissue samples were put on Techni Ice^®^ packs (Techni Ice, USA) in coolers for short-term storage, then transferred to a −80 °C freezer until sample preparation and analysis for THg and measurement of mRNA abundance profiles. Liver and kidney tissue samples were homogenized using an Omni General Laboratory Homogenizer (Omni International, Kennesaw, GA) to ensure uniform sample composition prior to mercury and mRNA analysis. Muscle tissue samples were not homogenized prior to mercury analysis, but muscle tissue sample collection location was standardized (Fig. S1).

### Mercury analysis

Total mercury wet mass fractions in fish tissues were measured by direct combustion atomic absorption spectrometry (AAS) with a DMA 80 Direct Mercury Analyzer (Milestone Scientific, Shelton, CT) using high purity oxygen as the carrier gas. Fish tissues were measured on a wet mass to mass (nanogram per gram; mass fraction) basis. Analysis of fish tissue and THg calculations were modeled after two previous methods described elsewhere (Hansen et al. 2016; Bryan et al. 2012). Briefly, approximately 100 mg of each fish tissue and control material sample was weighed out into pre-cleaned nickel weigh boats for analysis in the DMA 80. Mercury mass fractions were determined with external calibration curves (peak area versus ng Hg) prepared with NIST SRM^®^ 3133 Mercury Standard Solution (Lot No. 061204; Gaithersburg, MD). All measurements reported in the results and discussion sections are ng/g wet mass (wm) THg and referred to as THg in the rest of this paper.

NIST SRM^®^ 1946 Lake Superior Fish Tissue (Gaithersburg, MD), NIST in house quality control egg reference material (QC04ERM1), and instrumental blanks were bracketed in between blocks of eight to ten unknown fish tissue samples to confirm instrument calibration and monitor instrumental drift. QC04ERM1 and SRM 1946 were chosen as control materials because their THg mass fractions are in the low to high range of the calibration curves. Measurements of the control materials fell within the expanded uncertainty of their certificate values.

To ensure analytical homogeneity in muscle, liver, and kidney samples, repeat measurements were made from samples with enough material for each fish species (Table S3.) The results for the homogeneity of THg showed low within tissue variability in bonefish and trevally liver and muscle tissue with relative standard deviations under 10% (Table S3). The mean of the replicates of each fish liver and muscle tissue was used in the statistical analyses. However, the bonefish kidney tissue replicate measurements were not analytically homogenous for THg with relative standard deviations greater than 10% indicating great within sample heterogeneity (Table S3). To account for the kidney tissue THg variability, each bonefish kidney had two replicate measures and the mean was used for statistical comparisons.

### RNA extraction and cDNA formation

Total RNA was extracted from each fish liver and kidney sample using the reagents and protocol from the SV Total RNA Isolation System^™^ (Promega, Madison, WI). The purity of the RNA was evaluated using Thermo Scientific^®^ NanoDrop 2000 to determine the ratio of absorbance at 260 nm and 280 nm (A260/A280). RNA with a A260/A280 ratio of between 1.9 to 2.1 was deemed “pure” (Desjardins and Conklin 2010). Once the RNA was extracted from each liver and kidney tissue sample, cDNA was made by traditional PCR methods using SuperScript^®^ IV Reverse Transcriptase (ThermoFisher Scientific).

### Primer design and validation

Primers for β-actin (reference gene), MET, and TrxR were designed using MacVector with Assembler 15.1.2 (Cary, NC) and Primer3web version 4.0.0 (Untergasser et al. 2012; Koressaar and Remm 2007). Because gene sequences from the fish species in this study were not available, other fish (Actinopterygii) species were aligned to design primers in conserved regions in β-actin, MET, and TrxR sequences (Kurtz et al. 2019).

Candidate primer sets were tested and validated with fish samples by PCR and separated with gel electrophoresis. Bands were excised, cleaned, and sequenced by the ASGPB Genomics Laboratory at the University of Hawai‘i at Manoa. Optimal β-actin and MET primers were selected for bonefishes and trevallies, one TrxR1 primer set was validated for use in both genera, and one TrxR2 primer set produced a product in liver of the trevally species. Finally, the output was confirmed as either a β-actin, MET, or TrxR sequence through a BLAST search in GenBank (Table 1).

**Table 1:** qPCR Primers for β-actin, MET, TrxR, and TrxR2 designed for liver and kidney tissues in bonefish and trevally species

Gene Name	Primer Name	Primer sequence (5’→ 3’)	Product Size (base pair)	Tissue Used in for qPCR Assay
Thioredoxin Reductase 1	TrxR.4—FP	AAGTGGTTACCTGAGGAATGT	170	Bonefish liver and kidney; Trevally liver
	TrxR.4—RP	TGTGTGTCTGGACAGTGTGACT		
Thioredoxin Reductase 2	TrxR2.2—FP	GGAGGCCTTGCATGTTCC	104	Trevally liver
	TrxR2.2—RP	CCACCAAGACCCCACTTG		
Metallothionein	MT6—FP	ATGGATCCTTGCGACTGCTC	171	Trevally liver
	MT6—RP	GCTGGTGTCGCAGGTCTTC		
	MTF—FP	CCTCGCAGTGCACGGATT	148	Bonefish liver and kidney
	MTF—RP	TGGTGTCGCAGGTCTTCACT		
β-actin (reference)	BABL—FP	ACCTTGATTTTCATTGTGCTAGG	250	Bonefish liver and kidney
	BABL—RP	TCCACAGGTATGGAGTCATGC		
	BATL2—FP	CTTGATCTTCATGGTGGATGG	150	Trevally liver
	BATL2—RP	CTGCGGAATCCACGAGAC		

### qPCR protocol and relative mRNA abundance calculation

Metallothionein and thioredoxin reductase mRNA transcripts were measured in liver and kidney samples. Quantitative PCR (qPCR) performed with GoTaq^®^ PCR Master Mix (Promega) on a Mastercycler^®^ ep *realplex* (Eppendorf) detected MET and TrxR genes in fish liver and kidney samples, and relative mRNA concentrations were determined for both biomarkers using β-actin as the reference gene. β-actin, MET, and TrxR reactions for each fish sample were run in triplicate. Relative concentrations of MET and TrxR transcripts were calculated using the ΔΔC_q_ method from Bustin et al. (2009).

### Data analyses

Statistical analyses were performed using R (R Core Team 2019) and Microsoft Excel (Redmond, Washington). Figures were produced using R and Adobe Photoshop 23.5.1 Release. The catch location map (Fig. 1) was produced using QGIS 3.16.3 Hanover. Since THg did not meet the assumptions of a normal distribution, the values were normalized by log_10_ transformation and used in statistical analyses. Although a goal was to statistically compare THg in tissue from all four fish species, only round jaw bonefish and giant trevally results were included in the statistical analyses as the sample number of sharp jaw bonefish and bluefin trevally species were too few for statistical analysis. When homogeneity of variances was violated, nonparametric tests were employed. Significance levels were set at α = 0.05. Outlier analysis was performed on biomarker data with calculations from Hoaglin and Iglewicz (1987).

Species-specific relationships between THg in tissues and fork length along with calculated age were evaluated using separate regression analyses. Approximate ages were calculated using measured fork length and the parameters of the von Bertalanffy growth function (VBGF) recently published for round jaw bonefish (Donovan et al. 2015) and giant trevally (Pardee et al. 2021). To further assess Hg bioaccumulation, THg liver-to-muscle ratios were calculated for round jaw bonefish and giant trevally. Pearson’s correlations were used to evaluate pairwise trends in THg between tissue types for each species. Tissue differences in THg in round jaw bonefish and giant trevally were examined with one-way ANCOVAs accounting for the effect of fork length. Differences in muscle THg between round jaw bonefish and giant trevally were examined using a Mann-Whitney U test. Mean muscle THg in the two bonefish species and two trevally species were compared to other fish species that are commonly consumed in Hawai‘i with consumption guidelines based on the EPA/FDA criterion of 0.3 parts per million (ppm) THg in fish tissue. Mean muscle THg measurements for all fish species were converted to ppm for easier comparison and evaluation against the EPA/FDA and World Health Organization (0.5 ppm in fish tissue) criteria. The relationship between THg in fish tissues and the transcript levels of each biomarker in liver and kidney samples was examined using Pearson’s correlation analyses. Separate one-way ANOVAs were used to compare MET and TrxR mRNA expression in giant trevally liver, round jaw bonefish liver and round jaw bonefish kidney. Significant ANOVAs and ANCOVAs were followed with Bonferroni post-hoc tests.

## Results & discussion

### Total mercury in bonefish and trevally tissues

Total Hg accumulated differentially in round jaw bonefish tissues but did not show significant accumulation differences in giant trevally tissues. Round jaw bonefish (*n* = 19) mean kidney THg was 3.5 and 3.4 times greater than mean muscle and liver THg, respectively, controlling for fork length (One-way ANCOVA; *F* (2, 52) = 19.4, *p* < 0.001; Bonferroni post hoc tests: kidney and muscle *p* < 0.001; kidney and liver *p* < 0.001; Table 2). Similar findings were observed in golden grey mullet (*Liza aurata*) from the Ria de Aveiro Lagoon, Portugal (Mieiro et al. 2009) and spotted seatrout (*Cynoscion nebulosus*) from South Florida and Indian River Lagoon, Florida (Adams et al. 2010) where THg was greater in kidney compared to liver and muscle. In giant trevally, THg in muscle was not significantly different from THg in liver after controlling for the effect of fork length (one-way ANCOVA, *F* (1, 23) = 0.326, *p* = 0.574).

**Table 2:** Summary of morphometrics for samples from four fish species collected at Hawaiian fishing tournaments

Species	Common Name	*n*	Fork Length (cm)	Calculated Age (years)	THg muscle	THg liver	THg kidney	THg Liver: THg Muscle
*Albula glossodonta*	Round Jaw Bonefish	19	57.9 ± 1.5	12.0 ± 1.2	330 ± 45.1	340 ± 116	1156 ± 300	0.84 ± 0.12
*Albula virgata*	Sharp Jaw Bonefish	2	57 ± 2.8	9.7 ± 0.6	556 ± 208	1382 ± 78.7	3908 ± 1104	3.3 ± 1.5
*Caranx melampygus*	Bluefin Trevally	4	37.1 ± 2.5	3.4 ± 0.5	76.7 ± 8.9	63.0 ± 9.5		0.81 ± 0.04
*Caranx ignobilis*	Giant Trevally	13	51.1 ± 6.2	4.3 ± 1.0	188 ± 46.2	277 ± 107		1.3 ± 0.15

*n* represents the number of samples for each species. Calculated ages, THg (ng/g wm), and THg liver/muscle ratios in sampled tissues are shown as mean ± standard error (SE)

The mean THg liver/muscle ratio (0.84 ± 0.12; Table 2) calculated for round jaw bonefish in this study does not definitively indicate that THg uptake is increasing or decreasing. The mean THg liver/muscle ratio (1.3 ± 0.15; Table 2) in giant trevally from this study suggests that THg uptake is increasing. Mercury tissue accumulation and THg liver/muscle ratios in other fish species show varying results. Shorthorn sculpin (*Myoxocephalus scorpius*) collected in Alaska had significantly greater Hg accumulation in muscle tissue compared to liver and kidney (Harley et al. 2015). Cizdziel et al. (2003) measured THg in multiple tissues (skeletal muscle, liver, blood, gonad, brain, gill, and heart) of several freshwater fishes sampled from Lake Mead, USA and found greater Hg in liver in striped bass (*Roccus saxitilis*), channel catfish (*Ictalurus punctatus*), and bluefin tilapia (*Oreochromis aureus*) with THg liver/muscle ratios greater than or equal to 1 suggesting an increase in Hg uptake. In contrast, largemouth bass (*Micropterus salmoides*) had the greatest Hg in muscle with THg liver-to-muscle ratio of 0.52 indicating a decrease in Hg uptake; however, the authors cautioned using this ratio to determine rate of Hg uptake because muscle preferentially accumulates MeHg while the liver accumulates iHg (Cizdziel et al. 2003). Havelková et al. (2008) sampled fish species along the River Elbe and found fish in lightly contaminated areas preferentially accumulated Hg in muscle (lower THg liver/muscle ratios) while fish in heavily contaminated areas preferentially accumulated Hg in the liver (higher THg liver/muscle ratios). Cruz-Acevedo et al. (2019) also used THg liver/muscle ratios to show environmental contamination differences among marine fishes sampled off the coast of Mexico.

### Correlation of total mercury among tissues

**Fig. 2:**
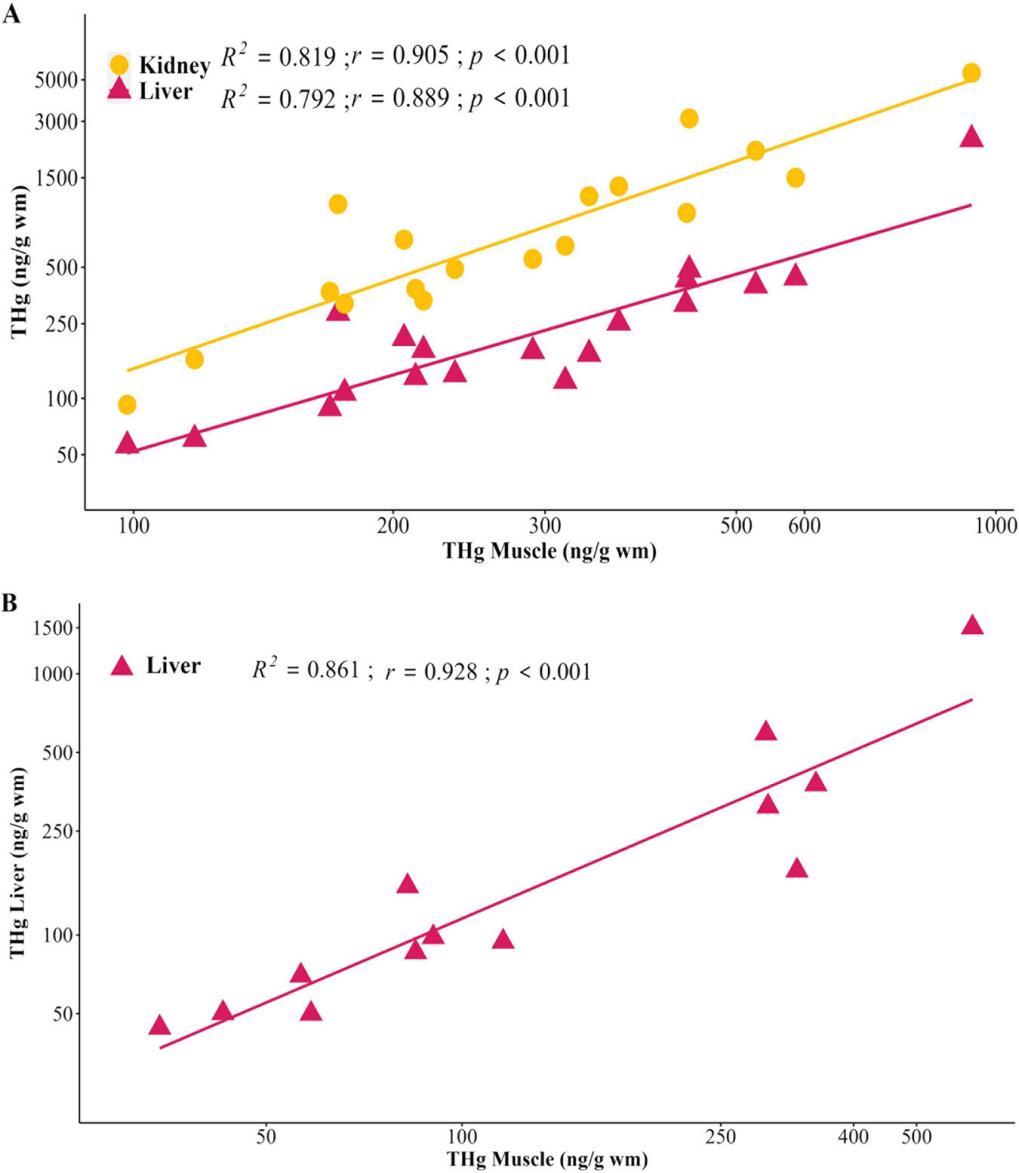
Correlation of THg among fish tissues: (**A**) round jaw bonefish THg muscle with THg kidney (dark yellow circles) and liver (magenta circles), respectively (**B**) giant trevally THg muscle with THg liver

Total Hg in giant trevally and round jaw bonefish showed tight pairwise trends between tissue types for each species. Round jaw bonefish muscle THg significantly positively correlated with liver and kidney THg (Fig. 2a); and THg liver and kidney were also significantly positively correlated (R^2^ = 0.837; *p* < 0.001). Similarly, giant trevally THg muscle significantly positively correlated with THg liver (Fig. 2b). These relationships suggest that THg accumulation in muscle could be indicative of other body organ THg accumulation and that nonlethal tissue sample collection could be used to characterize relative THg body burden. Results from the present study are consistent with previous studies. For example, THg in muscle was positively correlated with THg in liver in four freshwater fish species from Lake Mead, USA (striped bass, largemouth bass, channel catfish, blue tilapia (*Oreochromis aureus*)) (Cizdziel et al. 2003). Similarly, golden grey mullet had muscle THg positively correlated with THg in liver, kidney, as well as other tissues, including brain, blood, and gills (Mieiro et al. 2009).

### Relationship between total mercury, fish length, and calculated age

**Fig. 3:**
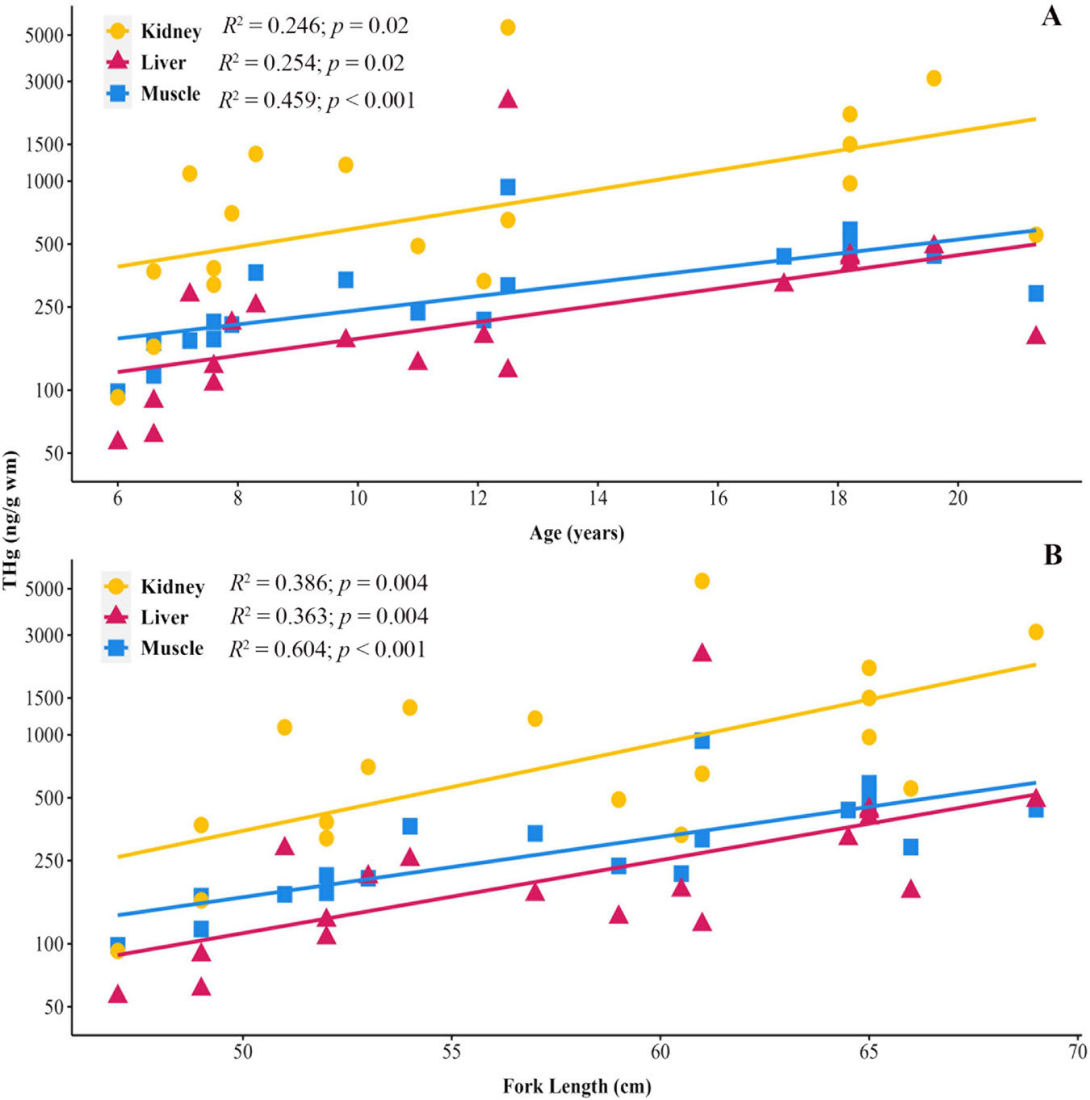
Relationship between round jaw bonefish tissue THg, age, and fish length: (**A**) regression analysis between calculated age and THg in tissues (kidney, dark yellow circle, *r* = 0.539; liver, magenta triangle, *r* = 0.543; muscle, blue square, *r* = 0.699) (**B**) regression analysis between fork length and THg in tissues (kidney, dark yellow circle, *r* = 0.621; liver, magenta triangle, *r* = 0.602; muscle, blue square, *r* = 0.777)

**Fig. 4:**
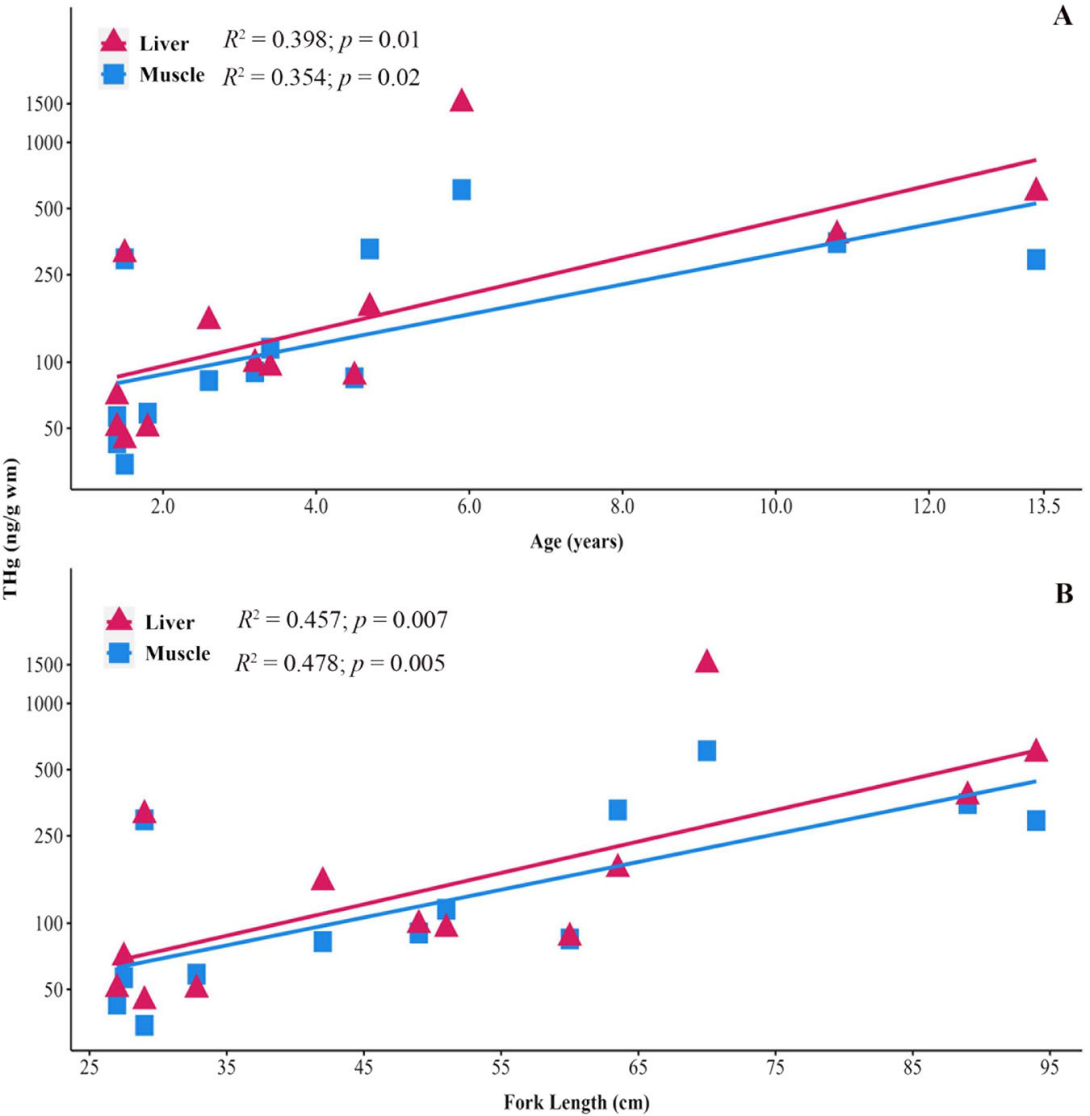
Relationship between giant trevally tissue THg, age, and fish length: (**A**) regression analysis between calculated age and THg in tissues (liver, magenta triangle, *r* = 0.670; muscle, blue square, *r* = 0.639) (**B**) regression analysis between fork length and THg in giant trevally tissues (liver, magenta triangle, *r* = 0.676; muscle, blue square, *r* = 0.691)

Species-specific regression analyses showed significant relationships between fork length and THg in round jaw bonefish and giant trevally tissues. Round jaw bonefish THg in muscle, liver, and kidney significantly positively correlated with fork length (Fig. 3b). In giant trevally tissues, THg in muscle and liver significantly correlated with fork length (Fig. 4b). Other studies have reported similar findings that Hg in muscle increases with fish fork length as observed in wahoo (*Acanthocybium solandri*), swordfish (*Xiphias gladius*), common dolphinfish (*Coryphaena hippurus*), black crappie (*Pomoxis nigromaculatus*), largemouth bass, and several tuna species (Kojadinovic et al. 2006; Chen et al. 2014; Sackett et al. 2013). Shorthorn sculpin collected in Alaskan waters had THg tissue (muscle, liver, kidney, and heart) positively correlate with age, which closely correlated with length (Harley et al. 2015).

In addition, the calculated age was also compared to THg in round jaw bonefish and giant trevally tissues. Calculated ages of round jaw bonefish positively correlated with THg in muscle, liver, and kidney tissues (Fig. 3a); and THg in giant trevally muscle and liver positively correlated with calculated age (Fig. 4a). These results suggest that Hg is bioaccumulating in round jaw bonefish and giant trevally tissues as these fishes grow and age. Fish length and age are not linear however but exhibit an asymptotic relationship where fish continue to age but do not continue to grow. If available, age and length measurements should be used together to assess bioaccumulation of THg in fish tissues.

### Muscle total mercury comparisons between study species and pelagic food fish species

**Fig. 5:**
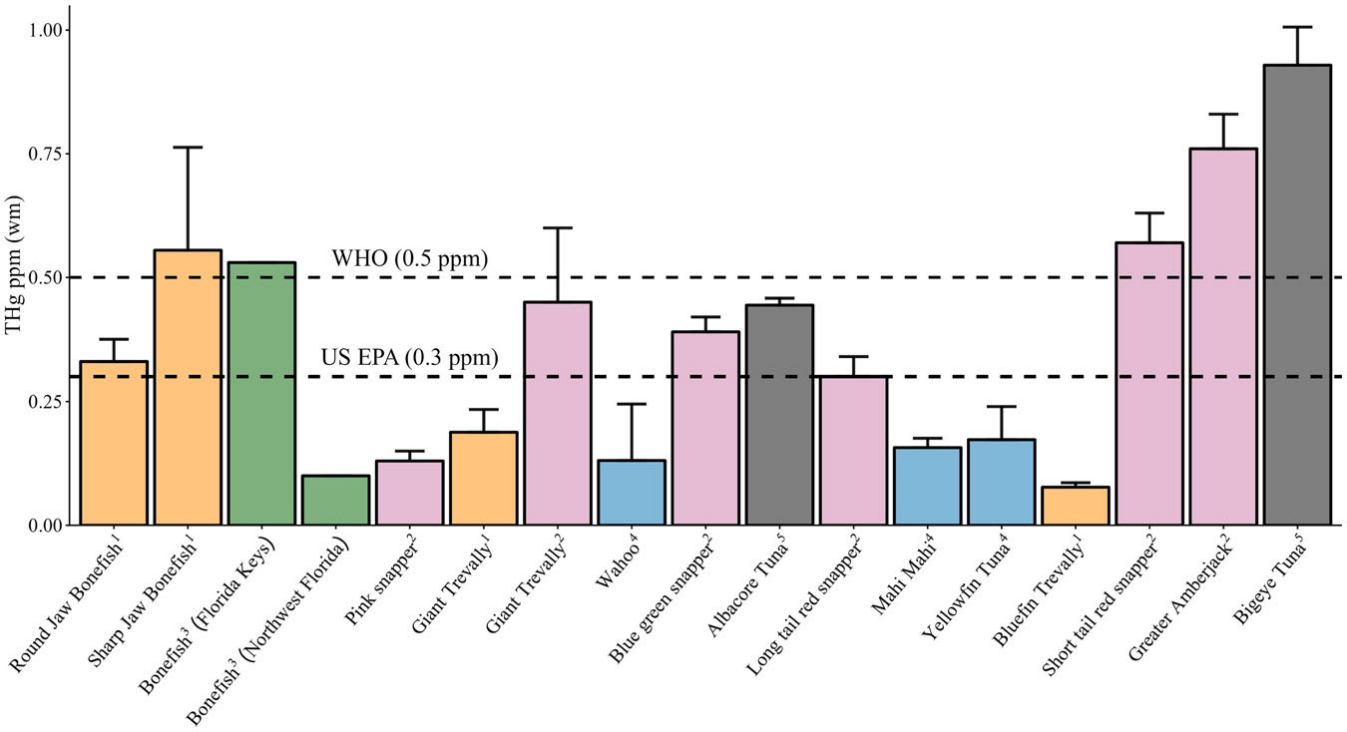
Total muscle mercury in food and recreational fish species in the Pacific Ocean and bonefish from Florida, USA. Total mean mercury and standard error in this study are compared to values in the same species or species inhabiting the same regional area of the Pacific Ocean reported in previous studies. Fish are ordered in ascending trophic position. Bars are color coded by study. Dotted lines represent US EPA THg criterion of 0.3 parts per million (ppm) in fish tissue and World Health Organization (WHO) criterion of 0.5 ppm in fish tissue (WHO 2011). ^**1**^Fishes from current study (orange). ^2^Fishes from Sackett et al. (2015) (pink). ^3^Adams et al. 2003 (green; did not report standard error). ^4^Buck et al. 2019 (blue). ^5^Fishes from Chen et al. (2014) (dark grey)

Mean muscle THg of the bonefish and trevally from this study were compared to THg reported for other Pacific fish species and Florida bonefish that are commonly caught and harvested for food and recreation (Fig. 5). Round and sharp jaw bonefish and Florida bonefish (*Albula vulpes*) have trophic positions of 3.3 and 3.7 respectively; pink snapper (*Pristipomoides filamentosus*) has a trophic position of 3.8 (Froese and Pauly 2021). Giant trevally have a trophic position of 4.2, and the remaining fish in Fig. 5 occupy trophic positions between 4.3 and 4.5 (Froese and Pauly 2021). Giant trevally (*n* = 15) also captured around the main Hawaiian Islands in a previous study had greater mean muscle THg and greater mean length compared to the giant trevally measured in the current study suggesting the giant trevally from the previous study were older with more bioaccumulated THg in muscle tissue (Fig. 5) (Sackett et al. 2015). The bluefin trevally mean muscle THg in this study was less than bigeye tuna (*n* = 75) (Chen et al. 2014) and the greater amberjack (*Seriola dumerili*) (*n* = 8) (Sackett et al. 2015) that occupy the same trophic position of 4.5. The average fork length of bluefin trevally captured in this study was 37.13 cm while the adult bluefin trevally can grow up to 80 cm (Pardee et al. 2021) also indicating bluefin trevally from this study are younger with less accumulated THg and not yet consuming the same size prey as adult bigeye tuna and greater amberjacks. Round jaw bonefish from this study had greater mean muscle THg than yellowfin tuna (*n* = 2) (Buck et al. 2019), long-tail red snapper (*Etelis coruscans*) (*n* = 30), mahi mahi (*Coryphaena hippurus*) (*n* = 6), wahoo (*Acanthocybium solandri*) (*n* = 2), pink snapper (*n* = 30) (Sackett et al. 2015), and bonefish sampled in northwest Florida (*n* = 4) (Adams et al. 2003). The sharp jaw bonefish from this study has greater mean muscle THg than all other fishes except for the long-tail red snapper (*n* = 30), greater amberjack (Sackett et al. 2015), and bigeye tuna (Chen et al. 2014) but similar values to bonefish (*n* = 13) sampled in the Florida Keys (Adams et al. 2003). However, our sample size of sharp jaw bonefish was small with two individuals and a large standard error. More sampling of sharp jaw bonefish is needed to determine if the mean muscle THg in sharp jaw bonefish from this study is representative of the rest of the species.

The U.S. Environmental Protection Agency (EPA) and Food and Drug Administration (FDA) provide a consumption advisory of commercial seafood species based on current Hg monitoring efforts (EPA 2022). The EPA reference dose for MeHg is 0.1 parts per million (ppm) and 0.3 ppm for Hg^2+^ (U.S. EPA 2001). Based on comprehensive review over several years of fish muscle Hg measurements, the EPA and FDA recommend consuming servings of albacore tuna, yellowfin tuna, snapper, and mahi mahi only once a week and avoid consuming bigeye tuna (EPA 2022). Hawaiʻi State Department of Health have similar guidelines and consumption advisories for these fish species (HEER Office 2019).

Round jaw and sharp jaw bonefish in this study had mean muscle THg that exceeded the US EPA mercury consumption guideline (0.3 ppm) and do not currently have consumptions advisories as the tuna and snapper species (Fig. 5). Giant trevally from this study did not exceed the US EPA mercury consumption guideline, but the giant trevally previously measured in Sackett et al. (2015) did exceed this guideline and currently do not have consumption advisories regarding mercury. These results suggest that continued Hg monitoring is warranted for both bonefish and trevally species caught in Hawaiian waters around O‘ahu and consumption advisories may need to be developed.

Interestingly, while round jaw bonefish feed at a lower trophic position than giant trevally, their THg muscle was greater (*U* = 62, *p* = 0.018) than THg muscle in giant trevally. This could be explained in this study by the difference in size (i.e., mean fork length) of each species and the differences in calculated ages. Giant trevally sexually mature around 60 cm (Sudekum 1991), while adult mature round jaw bonefish average 49.8 cm in length (Donovan et al. 2015). The mean calculated age of round jaw bonefish was 12.0 ± 1.2 years, and the mean calculated age of giant trevally was 4.3 ± 1.0 years. Round jaw bonefish in this study were on average larger and older than the giant trevally sampled resulting in greater THg in muscle tissue.

### Mercury related changes in liver and kidney mRNA abundance

**Fig. 6:**
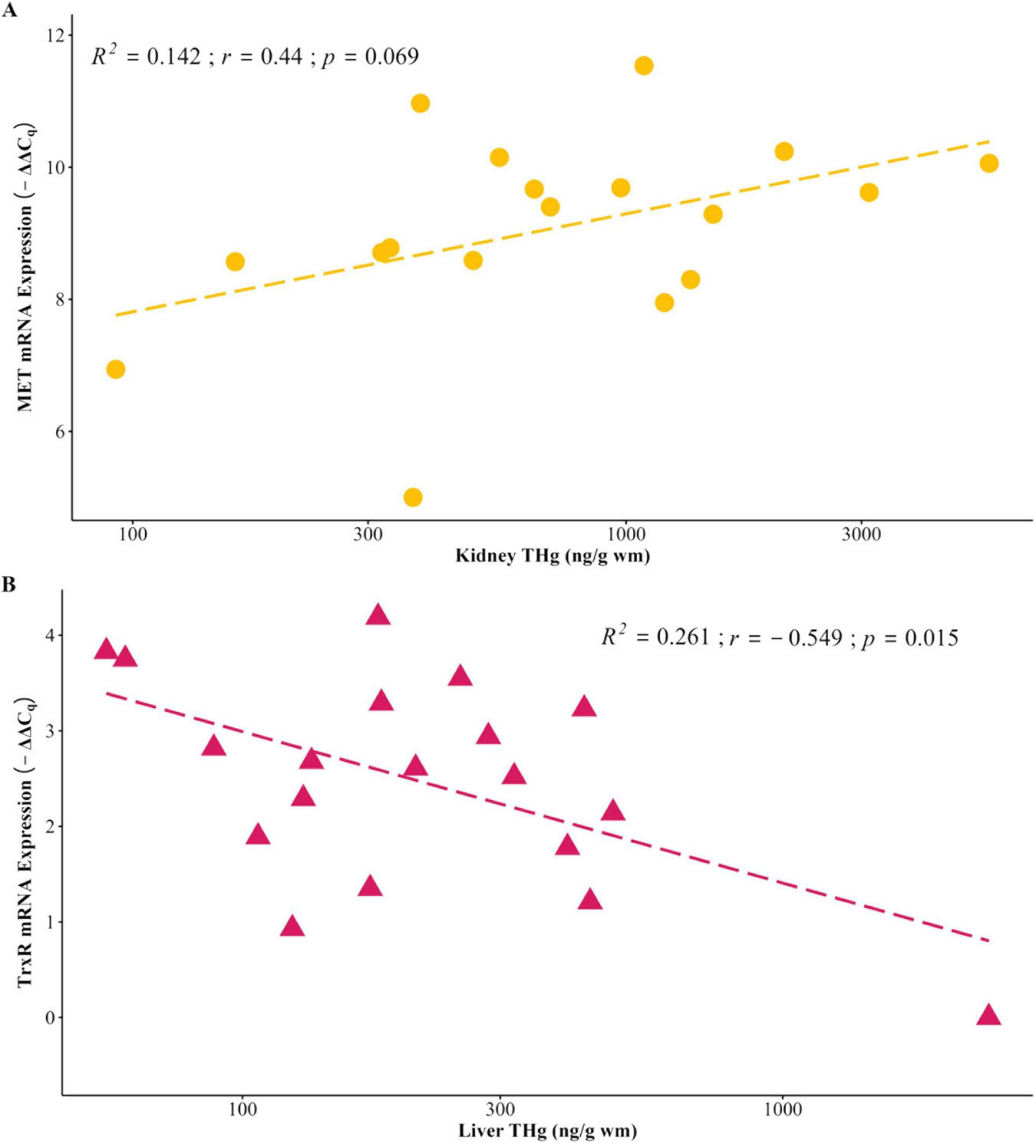
Relationship between mRNA expression and THg: (**A**) Correlation analysis between round jaw bonefish MET mRNA and THg in kidney and (**B**) Correlation analysis between round jaw bonefish TrxR1 mRNA and THg in liver

Metallothionein and TrxR mRNA transcript levels did not correlate to THg in giant trevally liver (*p* > 0.05), but TrxR mRNA transcript levels and round jaw bonefish liver THg did show a significant relationship. Currently, there are no other known studies that have assessed TrxR mRNA transcript levels in wild caught fish. Metallothionein mRNA transcript levels in round jaw bonefish kidney had a close to significant positive correlation with THg (*p* = 0.069) (Table 3; Fig. 6a). We observed a significant negative correlation between TrxR and THg in round jaw bonefish liver (Table 3; Fig. 6b). The lack of significant correlations between MET and TrxR mRNA transcripts and THg in giant trevally liver could be attributed to a small sample size (*n* = 13).

**Table 3:** Metallothionein, Thioredoxin Reductase 1, and Thioredoxin Reductase 2 mRNA transcript levels (Mean ± SD and Range) for samples from giant trevally and round jaw bonefish tissues

	Mean ± SD	Range	Mean ± SD	Range	R^2^	*p*-value to corresponding tissue THg
	fold change		-ΔΔCq			
Giant Trevally Liver (*n* = 13)						
MET	8.2 ± 6.1	1–19.2	2.6 ± 1.3	0–4.3	0.081	0.176
TrxR	41.4 ± 75.6	1.2–71.0	4.1 ± 1.9	0.2–8.2	0.086	0.822
TrxR2	17.4 ± 31.9	1–121	3.0 ± 1.7	0–6.9	0.069	0.653
Round Jaw Bonefish Liver (*n* = 19)						
MET	3444.3 ± 3513	763–6608	11.2 ± 1.2	9.6–12.7	0.024	0.462
TrxR	7.1 + 4.7	1–18.3	2.5 ± 1.1	0–4.2	0.261	0.015
Round Jaw Bonefish Kidney (*n* = 18)						
MET	804 ± 720	32–2978	9.1 ± 1.5	5–11.5	0.152	0.069
TrxR	3.8 ± 2.4	1–9.6	1.7 ± 0.9	0–3.3	0.013	0.387

The relationship between MET mRNA transcript and protein levels and Hg in fish species can vary (Berntssen et al. 2004; Mieiro et al. 2011; Walker et al. 2014; Guinot et al. 2012). In contrast to the unsignificant relationship between MET mRNA transcripts and liver THg observed in our study, MET mRNA transcript levels increased with increased MeHg exposure in zebrafish (*Danio rerio*) liver (Gonzalez et al. 2005). Navarro et al. (2009) observed a significant positive correlation between THg kidney and MET expression in feral carp populations with greater THg in carp kidney tissues from populations in greater contaminated areas than populations in less contaminated areas. Metallothionein mRNA expression in carp livers was relatively high among all populations with no correlation between increasing THg in liver tissue and MET expression (Navarro et al. 2009). Carp injected with total Hg ion showed significant induction of kidney MET expression but no significant induction of liver MET expression with relatively high MET expression before and after the induction experiment (Navarro et al. 2009). This suggests fish may have relatively high basal liver MET expression and low basal kidney MET expression that is induced in more contaminated environments. MET protein levels in fishes, rather than mRNA expression levels, are more commonly compared with Hg (Berntssen et al. 2004; Sinaie et al. 2010; Jebali et al. 2008; Gonzalez et al. 2005; Bebianno et al. 2007). Sea bass (*Dicentrarchus labrax*) injected with mercury chloride showed an increase in liver MET protein levels after 48 h of exposure (Jebali et al. 2008). In wild black scabbardfish (*Aphanopus carbo*), liver MET protein levels positively correlated to THg, while muscle MET protein levels inversely correlated to THg (Bebianno et al. 2007).

Very few vertebrate studies report how Hg exposure affects TrxR mRNA expression. For example, cell culture studies show that TrxR1 mRNA expression levels in HepG2 cells and rat brain cells increased with MeHg and Hg^2+^ concentrations (Fujimura and Usuki 2014; Branco et al. 2014). Several studies have examined the effects of Hg on TrxR activity, such as Hg exposure inhibited TrxR activity in zebra-seabream brain, liver, and kidney tissues (Branco et al. 2012) and mammalian cells (Carvalho et al. 2011). A larger sample size should be used for assessing TrxR mRNA transcript levels in wild caught fish in relation to Hg to further investigate the findings in the present study. Because TrxR is an important protein in cell functions for stress response, protein repair, and protection against oxidative damage, the significant relationship observed between round jaw bonefish tissues and TrxR mRNA transcript should be further investigated to evaluate possible negative health effects due to elevated Hg exposure in round jaw bonefish.

Exploring sublethal indicators of health impacts in environmentally exposed, non-traditional species is a challenge. The bonefish and trevally in this study were caught opportunistically in the waters around O‘ahu and not dosed with Hg in a controlled environment, so other factors are likely affecting MET and TrxR expression. Metallothionein functions to regulate essential metals, sequester non-essential metals, and protect against oxidative stress (Coyle et al. 2002). Metallothionein expression and protein levels vary depending on metals present in their habitat (Knapen et al. 2007), season (Olsson et al. 1987; Olsson et al. 1989), and water temperature (Nichols and Playle 2004; Serafim et al. 2002). Rainbow trout basal expression of MET isoforms have been shown to change seasonally in the liver and fluctuate based on an individual’s reproductive status (Olsson et al. 1990; Olsson et al. 1987). Thioredoxin reductase has a variety of functions in the cell, and any factors that affect the normal function of TrxR can impact the efficiency of cellular pathways. Thioredoxin reductase plays an important role in the cell by reducing selenium compounds to the active form (Se^2-^) and thioredoxin, which is needed for functioning in metabolic pathways, such as protein repair, redox signaling, and transcription regulation (Mustacich and Powis 2000; Xia et al. 2003; Holmgren and Lu 2010). If Hg accumulation is excessively elevated in tissue cells, Hg^2+^ sequesters Se^2-^ for Hg detoxification forming an inert HgSe (mercury selenide) crystal reducing the bioavailable amount of Se in the cell and preventing the formation of selenoproteins and enzymes (Seppänen et al. 2004; Ralston et al. 2008). The negative correlation observed between liver THg and TrxR1 mRNA expression in round jaw bonefish could indicate a lack of bioavailable Se^2-^ for TrxR formation. Although we did not observe many significant relationships between mRNA transcripts and THg in fish tissues, designing species specific primers for MET and TrxR and measuring the relative expressions for these transcripts provides a baseline for future work in monitoring how Hg exposure is affecting these culturally important fish species. Measuring protein levels of MET and TrxR in conjunction with mRNA expression and Hg may provide a clearer picture of how the regulation of these genes are affected by Hg.

### MET and TrxR mRNA differences between tissues and species

Metallothionein mRNA expression (–ΔΔC_q_) varied among round jaw bonefish kidney, liver, and giant trevally liver (One-way ANOVA, *F*(2, 47) = 165.7, *p* < 0.001). Each tissue differentially expressed metallothionein (Bonferroni post hoc test, *p* < 0.001 for all three comparisons). Round jaw bonefish liver had the greatest MET expression followed by round jaw bonefish kidney with giant trevally liver having the lowest MET expression. Thioredoxin reductase 1 mRNA expression (–ΔΔC_q_) also varied among the three fish tissues analyzed (One-way ANOVA, *F*(2, 47) = 12.9, *p* < 0.001). Giant trevally liver exhibited the greatest TrxR1 expression but was only significantly greater than TrxR1 expression in round jaw bonefish kidney (*p* < 0.001).

The basal MET and TrxR mRNA levels in these fish species’ tissues is not known, but METs are usually concentrated in liver, kidney, gills, and intestines of aquatic animals (Roesijadi, 1992), which may explain the relatively high mRNA expression in round jaw bonefish liver and kidney. However, the relatively low MET expression in giant trevally liver does not follow this pattern, and findings that show MET mRNA expression has high basal levels in fish liver (Navarro et al. 2009). Comparing the differences of gene expression between fish species is difficult because different habitats can have varying stressors, including mercury exposure, that will affect how fish MET mRNA abundance will vary. Because this is the first study to quantify relative MET and TrxR transcript levels in round jaw bonefish and giant trevally, we do not know if these levels are basal, elevated, or reduced. Thioredoxin reductase is believed to be in excess levels in the cell required to maintain normal levels of reduced Trx1 (Cheng et al. 2010). This excess of TrxR could be necessary to perform other functions, or in case of exposure to inhibition factors. When TrxR was inhibited by 90% in HeLa (human cell line) cells, Trx1 remained in a reduced state (Eriksson et al. 2009). Small interfering RNA knocked down TrxR1 by 90% in A549 (human epithelial cell) cells but did not seem to affect Trx activity or inhibit cell growth (Watson et al. 2008). Further work is needed to elucidate MET and TrxR expression patterns in fish.

## Conclusions

The results of this project provide the first examination of the effects of Hg on nearshore fish species around the Hawaiian Islands. Both round jaw bonefish and giant trevally exhibited Hg bioaccumulation in their tissues just as other studies have observed in several tuna species and fifteen additional commercially important offshore or pelagic Hawaiian fish species. Total Hg determined in giant trevally and round jaw bonefish muscle, liver, and kidney showed relationships that have not been published to date in these species. The relationship between THg in muscle tissue and internal fish organs in this study matched the relationship observed in several other fish species. Muscle THg in round jaw bonefish and giant trevally merit further monitoring. Mean muscle THg in round jaw bonefish exceeded WHO and US EPA fish tissue criteria for human consumption. Giant trevally mean muscle THg in this study was similar THg to commercial, pelagic fish species that have FDA consumption advisories. In addition to providing baseline mercury data for these nearshore fish species, the initial trends observed in this study suggest MET and TrxR expression may be useful biomarkers of Hg exposure in round jaw bonefish, but these relationships were not observed in giant trevally.

## Disclaimer

Certain commercial products and instruments are identified in this paper to adequately specify the experimental procedures. Such identification does not imply recommendation or endorsement by the National Institute of Standards and Technology. Nor does it imply that the items mentioned are the best for the intended purpose.

### Supplementary information

Supplementary Information
