# Supplementary material for: Mercury accumulation and biomarkers of exposure in two popular recreational fishes in Hawaiian waters
Source: Ecotoxicology. 2023 Jul 25;32(8):1010–23. doi: 10.1007/s10646-023-02684-1 (PMC10622350; doi:10.1007/s10646-023-02684-1)
Supplement: Supplementary file 1 — Supplementary Information [file 10646_2023_2684_MOESM1_ESM.pdf]

Supplemental information

Mercury Accumulation and Biomarkers of Exposure in Two Popular Recreational Fishes in Hawaiian Waters

Stephanie Shaw Holbert[1], Colleen E. Bryan[2], Keith Korsmeyer[1], and Brenda Jensen[1]
sdsholbert@gmail.com, colleen.bryan@nist.gov, kkorsmeyer@hpu.edu, and bjensen@hpu.edu

[1]College of Natural and Computational Sciences, Hawaiʻi Pacific University, Kaneohe, Hawaiʻi
[2]Chemical Sciences Division, National Institute of Standards and Technology, Charleston, South Carolina

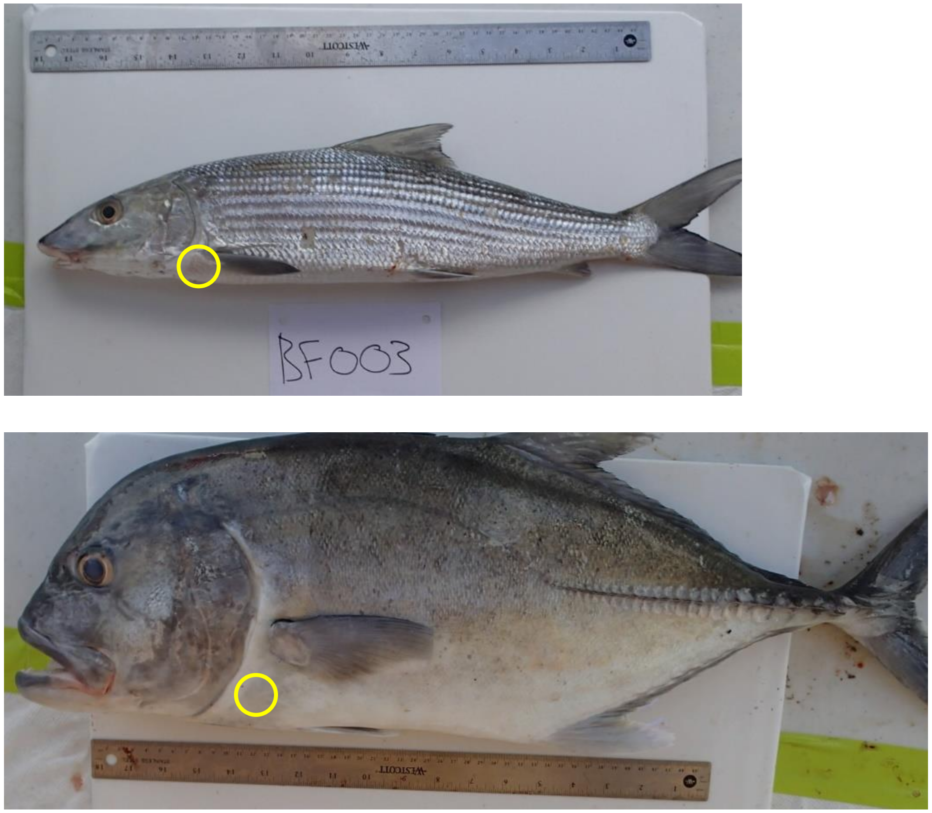

Figure S1. Location of muscle sample taken for mercury analysis in bonefish (top) and trevally (bottom).

Table S1. Bonefishes catch information, calculated age (Donovan et al., 2015), and total mercury mass fractions (ng/g wet mass) in tissue samples.

Sample ID	Fork Length (cm)	Calculated Age (years)	Sex	Species	Catch Date	Catch Location	Muscle THg	Liver THg	Kidney THg
B01	54.0	8.3	Female	Round	7-Nov-15	Hawaiʻi Kai	365.261	253.035	1350.686
B02	53.0	7.9	Male	Round	7-Nov-15	Hawaiʻi Kai	205.859	209.160	702.017
B03	47.0	6.0	Male	Round	6-Nov-15	Hawaiʻi Kai	98.292	55.978	92.434
B04	49.0	6.6	Female	Round	7-Nov-15	Kailua	168.883	88.508	370.053
B05	53.0	10.3	Female	Sharp	8-Nov-15	Kailua	261.344	1270.759	2347.537
B06	52.0	7.6	Female	Round	8-Nov-15	Kahuku	175.623	106.851	319.696
B07	60.5	12.1	Male	Round	6-Nov-15	Paiko's Lagoon	216.823	180.716	332.309
B08	59.0	11.0	Male	Round	6-Nov-15	Paiko's Lagoon	235.822	134.168	489.923
B09	52.0	7.6	Male	Round	13-Nov-15	Ewa Beach	212.301	129.603	382.990
B10	51.0	7.2	Female	Round	13-Nov-15	Ewa Beach	172.589	285.198	1086.620
B11	64.5	17.1	Male	Round	14-Nov-15	Ewa Beach	436.732	318.308	N/A
B12	49.0	6.6	Female	Round	14-Nov-15	Maleakahana	117.692	60.684	161.398
B13	65.0	18.2	Female	Round	15-Nov-15	ʻĀina Haina	437.973	429.035	975.460
B14	66.0	21.3	Female	Round	15-Nov-15	Laie	290.304	178.222	553.597
B15	61.0	12.5	Female	Round	21-Feb-16	South Shore	937.165	2406.071	5443.483
B16	69.0	19.6	Male	Round	20-Feb-16	ʻĀina Haina	440.939	485.173	3107.841
B17	57.0	9.8	Male	Round	20-Feb-16	South Shore	337.422	172.321	1196.221
B18	65.0	18.2	Male	Round	20-Feb-16	South Shore	526.492	399.473	2091.276
B19	65.0	18.2	Female	Round	20-Feb-16	Ewa Beach	585.756	440.020	1500.921
B20	61.0	12.5	Female	Round	20-Feb-16	Hawaiʻi Kai	316.594	123.668	651.468
B21	61.0	9.1	Female	Sharp	19-Feb-16	Ewa Beach	850.442	1493.407	5469.440

Table S2. Trevallies catch information, calculated age (Pardee et al., 2021), and total mercury mass fractions (ng/g wet mass) in tissue samples.

Sample ID	Fork Length (cm)	Calculated Age (years)	Sex	Species	Catch Date	Catch Location	Muscle THg	Liver THg
T01	51.0	3.4	Female	Giant	7-Nov-15	Hawaiʻi Kai	115.660	94.351
T02	49.0	3.2	Male	Giant	7-Nov-15	Hawaiʻi Kai	90.334	98.165
T03	42.0	2.6	Male	Giant	7-Nov-15	Hawaiʻi Kai	82.463	153.781
T04	60.0	4.5	Male	Giant	7-Nov-15	Hawaiʻi Kai	84.851	85.964
T05	94.0	13.4	Male	Giant	6-Nov-15	Kaneohe Bay	293.314	591.143
T06	29.0	1.5	Male	Giant	7-Nov-15	Kahuku	295.476	310.399
T07	29.0	1.5	Male	Giant	8-Nov-15	Kahuku	34.285	44.204
T08	32.8	1.8	Unknown	Giant	8-Nov-15	Kahuku	58.591	49.961
T09	33.0	2.8	Male	Bluefin	8-Nov-15	Kahuku	98.869	84.192
T10	27.5	1.4	Male	Giant	8-Nov-15	Kahuku	56.533	69.932
T11	27.0	1.4	Male	Giant	8-Nov-15	Kahuku	42.895	50.256
T12	63.5	4.7	Female	Giant	6-Nov-15	Kailua	327.422	176.597
T13	36.0	3.2	Unknown	Bluefin	8-Nov-15	Kahuku	69.357	51.341
T14	34.0	3.0	Male	Bluefin	8-Nov-15	Kahuku	86.729	78.413
T15	45.5	4.8	Male	Bluefin	8-Nov-15	Eastside Diamondhead	51.857	37.873
T16	70.0	5.9	Male	Giant	14-Nov-15	Hauula	609.428	1504.964
T17	89.0	10.8	Male	Giant	20-Feb-16	Kahuku	350.119	377.588

Table S3. Tissue Replicate Study to Determine Homogeneity within Sample Type

Several aliquots within samples of each tissue were measured to test homogeneity within sample type. Unlike liver and kidney tissue samples, muscle samples were not homogenized, so this replicate study was needed for muscle homogeneity assurance. However, liver and kidney mercury homogeneity were also tested to confirm liver and kidney samples were properly homogenized. Acceptable percent relative standard deviation was set at 10% or below. (GT = giant trevally; TC = trevally control; BF = bonefish)

	**Replicate [Hg] (ng/g wet mass)**						**Mean**	**STD**	**%RSD**
**Sample**	**1**	**2**	**3**	**4**	**5**	**6**			
GT17 Fin Muscle	364.796	345.208	359.345	326.758	354.486		350.119	14.912	4.259
GT17 Middle Muscle	312.724	344.363	323.522	311.941	305.517		319.614	15.271	4.778
GT17 Back Muscle	374.911	375.660	316.371	384.127	402.244		370.663	32.285	8.710
TC LF Muscle	69.017	69.274	68.089				68.794	0.623	0.906
TC LM Muscle	71.302	72.096	73.955				72.451	1.362	1.880
TC LB Muscle	81.711	80.807	77.820	76.006			79.086	2.642	3.341
TC RF Muscle	75.513	70.653	68.714	69.243			71.031	3.099	4.362
TC RM Muscle	73.346	72.717	72.272	74.412			73.187	0.928	1.268
TC RB Muscle	78.740	78.161	78.863	76.988			78.188	0.857	1.096
GT01 Liver	93.834	94.874	92.989	95.705			94.351	1.187	1.258
GT09 Liver	90.836	83.574	82.589	79.768			84.192	4.714	5.599
GT17 Liver	438.055	359.985	386.340	332.703	388.951	359.490	377.587	36.095	9.559
BF01 Muscle	390.276	359.951	375.965	347.103	353.008		365.261	17.666	4.837
BF14 Muscle	291.703	284.895	294.314				290.304	4.863	1.675
BF21 Muscle	877.544	897.489	803.158	855.951	818.070		850.442	39.566	4.652
BF01 Liver	252.993	253.077					253.035	0.060	0.024
BF14 Liver	169.605	175.888	183.081	184.316			178.222	6.842	3.839
BF21 Liver	1663.192	1452.309	1352.737	1505.390			1493.407	129.676	8.683
BF06 Kidney	294.988	335.554	305.264	342.981			319.696	23.184	7.252
BF17 Kidney	1025.161	945.681	1110.918	1252.623	1646.722		1196.221	276.333	23.101
BF21 Kidney	2922.943	7043.710	7102.839	7325.235	2952.471	5469.440	5469.440	2313.551	42.300

Table S4. MET, TrxR1, and TrxR2 mRNA Expression in Bonefish and Trevally Tissues.

		mRNA Expression (fold change relative to baseline)			mRNA Expression (-ΔΔCq relative to baseline)		
Fish ID	Species	MET	TrxR1	TrxR2	MET	TrxR1	TrxR2
TCL	Bigeye Trevally	5.464	13.74	5.098	2.45	3.78	2.35
T01L	Giant Trevally	8.112	20.39	4.112	3.02	4.35	2.04
T02L	Giant Trevally	11.55	7.260	20.53	3.53	2.86	4.36
T03L	Giant Trevally	3.031	47.18	9.849	1.6	5.56	3.3
T04L	Giant Trevally	18.25	38.59	19.29	4.19	5.27	4.27
T05L	Giant Trevally	6.364	8.754	1	2.67	3.13	0
T06L	Giant Trevally	15.14	71.01	17.27	3.92	6.15	4.11
T07L	Giant Trevally	2.479	6.635	4	1.31	2.73	2
T08L	Giant Trevally	2.639	10.85	4.925	1.4	3.44	2.3
T09L	Bluefin Trevally	6.190	1	3.364	2.63	0	1.75
T10L	Giant Trevally	6.543	284.05	121.1	2.71	8.15	6.92
T11L	Giant Trevally	1	1.149	3.387	0	0.2	1.76
T12L	Giant Trevally	6.589	13.45	3.340	2.72	3.75	1.74
T13L	Bluefin Trevally	18.00	99.73	19.16	4.17	6.64	4.26
T14L	Bluefin Trevally	16.91	13.18	19.97	4.08	3.72	4.32
T15L	Bluefin Trevally	7.260	5.028	8.815	2.86	2.33	3.14
T16L	Giant Trevally	5.540	9.849	12.73	2.47	3.3	3.67
T17L	Giant Trevally	19.16	18.38	5.063	4.26	4.2	2.34
BF01L	Round Jaw Bonefish	762.7	11.71	—	9.575	3.55	—
BF02L	Round Jaw Bonefish	1428.2	6.105	—	10.48	2.61	—
BF03L	Round Jaw Bonefish	2352.5	14.22	—	11.2	3.83	—
BF04L	Round Jaw Bonefish	1038.3	7.062	—	10.02	2.82	—
BF05L	Sharp Jaw Bonefish	1	13.09	—	0	3.71	—
BF06L	Round Jaw Bonefish	3327.0	3.706	—	11.7	1.89	—
BF07L	Round Jaw Bonefish	1082.4	9.781	—	10.08	3.29	—
BF08L	Round Jaw Bonefish	6608.0	6.409	—	12.69	2.68	—
BF09L	Round Jaw Bonefish	3875.1	4.891	—	11.92	2.29	—
BF10L	Round Jaw Bonefish	867.1	7.674	—	9.76	2.94	—
BF11L	Round Jaw Bonefish	996.0	5.736	—	9.96	2.52	—
BF12L	Round Jaw Bonefish	3444.3	13.45	—	11.75	3.75	—
BF13L	Round Jaw Bonefish	6382.9	9.383	—	12.64	3.23	—
BF14L	Round Jaw Bonefish	15286.8	18.25	—	13.9	4.19	—
BF15L	Round Jaw Bonefish	1323.4	1	—	10.37	0	—
BF16L	Round Jaw Bonefish	1438.2	4.408	—	10.49	2.14	—
BF17L	Round Jaw Bonefish	6208.4	2.549	—	12.6	1.35	—
BF18L	Round Jaw Bonefish	5556.7	3.434	—	12.44	1.78	—
BF19L	Round Jaw Bonefish	1964.6	2.313	—	10.94	1.21	—
BF20L	Round Jaw Bonefish	1499.2	1.905	—	10.55	0.93	—
BF21L	Sharp Jaw Bonefish	1.266	14.62	—	0.34	3.87	—
BF01K	Round Jaw Bonefish	315.2	3.364	—	8.3	1.75	—
BF02K	Round Jaw Bonefish	675.6	6.543	—	9.4	2.71	—
BF03K	Round Jaw Bonefish	122.8	5.028	—	6.94	2.33	—
BF04K	Round Jaw Bonefish	32	8.168	—	5	3.03	—
BF05K	Sharp Jaw Bonefish	18.51	17.88	—	4.21	4.16	—
BF06K	Round Jaw Bonefish	418.8	1.042	—	8.71	0.06	—
BF07K	Round Jaw Bonefish	439.6	3.506	—	8.78	1.81	—
BF08K	Round Jaw Bonefish	385.3	3.364	—	8.59	1.75	—
BF09K	Round Jaw Bonefish	2005.9	1.765	—	10.97	0.82	—
BF10K	Round Jaw Bonefish	2977.7	1.919	—	11.54	0.94	—
BF12K	Round Jaw Bonefish	380.0	3.972	—	8.57	1.99	—
BF13K	Round Jaw Bonefish	826.0	4.891	—	9.69	2.29	—
BF14K	Round Jaw Bonefish	1136.2	9.646	—	10.15	3.27	—
BF15K	Round Jaw Bonefish	1067.5	3.784	—	10.06	1.92	—
BF16K	Round Jaw Bonefish	786.9	2.346	—	9.62	1.23	—
BF17K	Round Jaw Bonefish	247.3	4	—	7.95	2	—
BF18K	Round Jaw Bonefish	1209.3	2.014	—	10.24	1.01	—
BF19K	Round Jaw Bonefish	626.0	1	—	9.29	0	—
BF20K	Round Jaw Bonefish	814.6	2.497	—	9.67	1.32	—
BF21K	Sharp Jaw Bonefish	1	18.25	—	0	4.19	—
